# Exploring missed opportunities for influenza vaccination and influenza vaccine co-administration patterns among Italian older adults: a retrospective cohort study

**DOI:** 10.1093/eurpub/ckad155

**Published:** 2023-08-25

**Authors:** Alexander Domnich, Andrea Orsi, Matilde Ogliastro, Carlo-Simone Trombetta, Marianna Scarpaleggia, Chiara Ceccaroli, Carla Amadio, Anna Raffo, Luca Berisso, Alla Yakubovich, Giacomo Zappa, Daniela Amicizia, Donatella Panatto, Giancarlo Icardi

**Affiliations:** Hygiene Unit, San Martino Policlinico Hospital—IRCCS for Oncology and Neurosciences, Genoa, Italy; Hygiene Unit, San Martino Policlinico Hospital—IRCCS for Oncology and Neurosciences, Genoa, Italy; Department of Health Sciences (DISSAL), University of Genoa, Genoa, Italy; Interuniversity Research Center on Influenza and Other Transmissible Infections (CIRI-IT), Genoa, Italy; Department of Health Sciences (DISSAL), University of Genoa, Genoa, Italy; Department of Health Sciences (DISSAL), University of Genoa, Genoa, Italy; Department of Health Sciences (DISSAL), University of Genoa, Genoa, Italy; Local Health Unit 3 (ASL3), Genoa, Italy; Local Health Unit 3 (ASL3), Genoa, Italy; Local Health Unit 3 (ASL3), Genoa, Italy; Local Health Unit 3 (ASL3), Genoa, Italy; Local Health Unit 3 (ASL3), Genoa, Italy; Local Health Unit 3 (ASL3), Genoa, Italy; Department of Health Sciences (DISSAL), University of Genoa, Genoa, Italy; Interuniversity Research Center on Influenza and Other Transmissible Infections (CIRI-IT), Genoa, Italy; Regional Health Agency of Liguria (ALiSa), Genoa, Italy; Department of Health Sciences (DISSAL), University of Genoa, Genoa, Italy; Interuniversity Research Center on Influenza and Other Transmissible Infections (CIRI-IT), Genoa, Italy; Hygiene Unit, San Martino Policlinico Hospital—IRCCS for Oncology and Neurosciences, Genoa, Italy; Department of Health Sciences (DISSAL), University of Genoa, Genoa, Italy; Interuniversity Research Center on Influenza and Other Transmissible Infections (CIRI-IT), Genoa, Italy

## Abstract

**Background:**

Missed opportunities constitute a main driver of suboptimal seasonal influenza vaccination (SIV) coverage in older adults. Vaccine co-administration is a way to reduce these missed opportunities. In this study, we quantified missed opportunities for SIV, identified some of their socio-structural correlates and documented SIV co-administration patterns.

**Methods:**

In this registry-based retrospective cohort study, we verified the SIV status of all subjects aged ≥65 years who received at least one dose of coronavirus disease 2019 (COVID-19), pneumococcal or herpes zoster vaccines during the 2022/23 influenza season. The frequency of concomitant same-day administration of SIV with other target vaccines was also assessed.

**Results:**

Among 41 112, 5482 and 3432 older adults who received ≥1 dose of COVID-19, pneumococcal and herpes zoster vaccines, missed opportunities for SIV accounted for 23.3%, 5.0% and 13.2%, respectively. Younger, male and foreign-born individuals were generally more prone to missing SIV. The co-administration of SIV with other recommended vaccines was relatively low, being 11.0%, 53.1% and 17.1% in COVID-19, pneumococcal and herpes zoster cohorts, respectively.

**Conclusions:**

A sizeable proportion of older adults who received other recommended vaccines during the last influenza season did not receive SIV. This share of missed opportunities, which are subject to some social inequalities, may be addressed by increasing vaccine co-administration rates and implementing tailored health promotion interventions.

## Introduction

Seasonal influenza is a top-ranked infectious disease in terms of its high incidence and high mortality rates.^1^ Seasonal influenza vaccination (SIV) is among the most effective public health measures able to reduce the heavy socio-economic burden of this disease, and older adults are among the principal target groups for annual SIV.^2^

Despite the proven effectiveness of SIV and policy recommendations, coverage rates in most industrialized countries are still low.^3^^,^^4^ Alongside increasing community demands for and access to SIV, interventions that target healthcare providers may increase SIV uptake.^5^ Indeed, ineffective provider practices lead to missed opportunities for vaccination (MOV).^6^ MOV occur in all primary care settings and contribute to low vaccination rates.^7^ A missed opportunity for SIV (MOSIV) can be defined as any contact with healthcare services by a subject who is eligible for SIV which does not result in that subject actually receiving SIV.^8^ Identifying the determinants of MOSIV among individuals at high risk of influenza-related complications, despite one or more healthcare visits during the influenza season, is essential in order to improve SIV uptake.^9^

Vaccine co-administration, defined as the administration of two or more vaccines during the same visit, may reduce MOSIV.^10^ Influenza, pneumococcal disease, herpes zoster (HZ) and coronavirus disease 2019 (COVID-19) vaccines are the ‘big four’ vaccines recommended for seniors in order to ensure healthy ageing.^11^ Severity of these four vaccine-preventable diseases is higher in older adults and may result in sequelae, such as exacerbation of underlying health conditions, onset of frailty, increased long-term care dependency and reduced quality of life.^11^ Several randomized controlled trials have established that SIV can be co-administered with several vaccines—13-valent^12^ and 20-valent^13^ conjugate, 23-valent polysaccharide^14^ pneumococcal, live attenuated^15^ and adjuvanted subunit^16^ HZ and COVID-19^10^ vaccines—without causing clinically significant interference in terms of both immunogenicity and safety. In addition to reducing MOV, co-administration strategies may be more cost-effective than sequential vaccination.^17^ Finally, if for some reason a healthcare provider or patient prefers not to administer vaccines simultaneously, they may schedule vaccination during the next visit.

In Italy, SIV is recommended for all older adults and is free of charge.^18^ Most vaccinations are performed by general practitioners (GPs), who are remunerated for each SIV dose administered.^19^ However, SIV coverage among Italian seniors remains insufficient (e.g. 56.7% in the 2022/23 season),^20^ and far from the recommended minimum target of 75%.^18^

Attitudes towards adult vaccinations may be interrelated.^21^ For instance, previous SIV receipt has been reported to increase the likelihood of COVID-19^22^ and pneumococcal^23^ vaccinations. On the other hand, both uptake and awareness of being eligible for vaccination are higher for SIV than for pneumococcal and HZ vaccines.^24^ It is therefore likely that SIV-eligible subjects who receive other vaccines during the winter can be easily reached by interventions aimed at reducing MOSIV. While previous research has mostly focused on SIV as an enabler for other adult vaccinations,^10^^,^^22^^,^^23^ little is known about SIV status and its correlates in adults who have received pneumococcal, HZ and COVID-19 vaccines (henceforth referred to as target vaccines). In this study, we aimed to address two research questions. First, what is the frequency of 2022/23 SIV receipt among older adults who have been administered other recommended vaccines and which factors are associated with SIV? Second, to what extent are both SIV and target vaccines co-administered?

## Methods

### Study population and procedures

In this retrospective cohort, the study population consisted of older adults aged ≥65 years residing in the catchment area of Local Health Unit (LHU) 3, which covers most of the territory of the Metropolitan City of Genoa (Liguria, north-western Italy). As of 2022, the total number of adults aged ≥65 years was 196 762 (28.96% of the total population).

All individuals aged ≥65 years who had received at least one dose of the available pneumococcal, HZ and/or COVID-19 vaccines (either before or after SIV) during the 2022/23 winter season were potentially eligible. Otherwise, no exclusion criteria were set. Individual vaccination records from 1 October 2022 to 28 February 2023 were retrieved from an electronic vaccination registry of LHU 3. The study period selected was consistent with the Italian recommendations for the 2022/23 influenza season,^18^ which advise that the SIV campaign should start in early October and continue for the entire season. On a *post hoc* evaluation, we found only 17 SIV doses administered outside the study period. Finally, each subject was matched to his or her SIV status, if reported in the same registry.

This study was approved by the Ethics Committee of the Liguria Region (# 166/2023 ID 13094).

### Study variables

There were two study outcomes. First, MOSIV was defined as the proportion of subjects who had received at least one dose of any target vaccine, but had not been vaccinated with SIV, during the entire study period. Second, co-administration of SIV and target vaccines was defined as the proportion of subjects who had received both SIV and any target vaccine on the same day in relation to the total number of subjects who had received SIV and any target vaccine at any time during the study period.

Sex (female vs. male), age (continuous) and nationality (Italian vs. foreign-born) were the independent variables of interest. Moreover, to adjust for possible differences in the availability of single vaccines, the models were adjusted for the month of vaccination. Entry of all study variables in the vaccination registry was mandatory, and no missing data were found.

### Statistical analysis

Categorical variables were reported as proportions with exact Clopper-Pearson’s 95% confidence intervals (CIs) and continuous variables as means with standard deviations. The crude effect size was expressed as an odds ratio (OR), which was calculated through simple logistic regression. Multivariable logistic regression was finally applied in order to establish adjusted ORs (aORs) on the association between 2022/23 SIV status and the independent variables of interest. Potential interaction effects were tested in all models. Considering that the three target vaccines and associated campaigns differ by several characteristics (e.g. vaccine roll-out, availability, perceived importance), three separate models were constructed.

All analyses were performed in R stat packages v. 4.1.0 (R Core Team, Vienna, Austria).

## Results

A total of 5482, 3432 and 41 112 adults received ≥1 dose of pneumococcal, HZ and COVID-19 vaccines, respectively. Subjects in all cohorts were on average in their 70s, females slightly prevailed and at least 97% were natives. Most vaccinations were performed between October and November 2022. MOSIV accounted for 5.0% (95% CI: 4.5–5.6%), 13.2% (95% CI: 12.1–14.3%) and 23.3% (95% CI: 22.9–23.8%) in the pneumococcal, HZ and COVID-19 cohorts, respectively (table 1).

**Table 1:** Characteristics of the study participants

Variable	Level	Cohort		
		Pneumococcal (*n *=* *5482)	Herpes zoster (*n *=* *3432)	COVID-19 (*n *=* *41 112)
Sex	Female, % (*n*)	54.0 (2960)	50.8 (1743)	54.6 (22 444)
	Male, % (*n*)	46.0 (2522)	49.2 (1689)	45.4 (18 668)
Age (years), mean (SD)	Total	74.5 (7.5)	71.6 (6.8)	77.3 (7.6)
	Female	74.8 (7.8)	71.5 (6.9)	77.9 (7.9)
	Male	74.2 (7.1)	71.6 (6.7)	76.6 (7.1)
Nationality	Italian, % (*n*)	98.1 (5377)	96.9 (3327)	98.2 (40 386)
	Foreign-born, % (*n*)	1.9 (105)	3.1 (105)	1.8 (726)
Month of target vaccination	Oct 2022, % (*n*)	16.7 (917)	26.7 (915)	37.1 (15 240)
	Nov 2022, % (*n*)	47.0 (2574)	26.3 (901)	36.2 (10 774)
	Dec 2022, % (*n*)	19.5 (1067)	21.3 (731)	19.5 (7997)
	Jan 2023, % (*n*)	9.9 (541)	14.0 (482)	12.7 (5233)
	Feb 2023, % (*n*)	7.0 (383)	11.7 (403)	4.5 (1868)
Influenza vaccination	Yes, % (*n*)	95.0 (5207)	86.8 (2980)	76.7 (31 517)
	No, % (*n*)	5.0 (275)	13.2 (452)	23.3 (9595)
Month of influenza vaccination	Oct 2022, % (*n*)	26.5 (1379)	27.1 (931)	27.9 (8791)
	Nov 2022, % (*n*)	61.4 (3197)	57.0 (1700)	57.8 (18 203)
	Dec 2022, % (*n*)	11.2 (584)	10.7 (318)	12.9 (4061)
	Jan 2023, % (*n*)	0.8 (42)	0.9 (26)	1.2 (380)
	Feb 2023, % (*n*)	0.1 (5)	0.2 (5)	0.3 (82)

Note: SD, standard deviation.

As shown in table 2, in both pneumococcal and HZ cohorts each 1-year increase in age was associated with 6% (*P *<* *0.001) higher odds of receiving SIV. In these two cohorts, SIV receipt was also substantially lower among foreign-born individuals, while no effect of sex was observed. No significant interaction terms were found in the pneumococcal and HZ cohorts. Conversely, in the COVID-19 cohort, two significant interactions were established in the multivariable model. First, while females were more likely to be vaccinated (*P *<* *0.001), SIV receipt in males increased with age (*P* for interaction < 0.001). The main effect of age, however, was not significant (*P *=* *0.25). Second, foreign-born individuals had 42% lower odds (*P *<* *0.001) of receiving SIV. SIV receipt by nationality, however, depended on sex (*P* for interaction 0.046), with foreign-born men showing a lower frequency of SIV receipt.

**Table 2:** Correlates of 2022/23 seasonal influenza vaccination, by cohort

Variable	Level	2022/23 seasonal influenza vaccination			
		Yes, % (*n*)	No, % (*n*)	OR (95% CI)	**aOR (95% CI)** ^a^
Pneumococcal vaccination					
Sex	Female	94.8 (2805)	5.2 (155)	Ref	Ref
	Male	95.2 (2402)	4.8 (120)	1.11 (0.87–1.41)	1.09 (0.85–1.39)
Age, years	Mean (SD)	74.6 (7.5)	71.4 (6.4)	1.07 (1.05–1.09)^b^***	1.06 (1.04–1.08)^b^***
Nationality	Italian	95.1 (5112)	4.9 (265)	Ref	Ref
	Foreign-born	90.5 (95)	9.5 (10)	0.49 (0.25–0.96)*	0.64 (0.32–1.25)
Herpes zoster vaccination					
Sex	Female	86.9 (1514)	13.1 (229)	Ref	Ref
	Male	86.8 (1466)	13.2 (223)	0.99 (0.82–1.21)	0.95 (0.78–1.17)
Age, years	Mean (SD)	71.9 (6.9)	69.4 (5.7)	1.07 (1.05–1.09)^b^***	1.06 (1.05–1.08)^b^***
Nationality	Italian	87.4 (2907)	12.6 (420)	Ref	Ref
	Foreign-born	69.5 (73)	30.5 (32)	0.33 (0.21–0.51)***	0.37 (0.24–0.57)***
COVID-19 vaccination					
Sex	Female	75.9 (17 036)	24.1 (5408)	Ref	Ref
	Male	77.6 (14 481)	22.4 (4187)	1.10 (1.05–1.15)***	0.38 (0.23–0.61)***
Age, years	Mean (SD)	77.4 (7.5)	77.2 (7.9)	1.00 (1.00–1.01)^b^*	1.00 (0.99–1.00)^b^
Nationality	Italian	76.9 (31 058)	23.1 (9328)	Ref	Ref
	Foreign-born	63.2 (459)	36.8 (267)	0.52 (0.44–0.60)***	0.58 (0.48–0.69)***
Sex × age	–	–	–	1.01 (1.01–1.02)***	1.01 (1.01–1.02)***
Sex × nationality	–	–	–	0.68 (0.49–0.95)*	0.71 (0.51–0.99)*

Note: OR, odds ratio; aOR, adjusted odds ratio; SD, standard deviation.

a Adjusted for age, sex, nationality and month of target vaccination.

b 1-year increase.

* *P *<* *0.05,

*** *P *<* *0.001.

Among subjects who received both the 2022/23 SIV and a target vaccine, co-administration patterns varied by cohort. In the pneumococcal cohort, 53.1% (95% CI: 51.7–54.5%) of individuals were vaccinated on the same day. By contrast, co-administration occurred less frequently in the HZ (17.1%; 95% CI: 15.8–18.5%) and COVID-19 (11.0%; 95% CI: 10.7–11.4%) cohorts.

## Discussion

Our study showed that MOSIV was a frequent phenomenon among Italian older adults who were vaccinated with other target vaccines during the 2022/23 influenza season. Indeed, a total of 10 322 vaccination visits resulted in MOSIV, which corresponds to 5.2% of the total senior population. The study area has one of the highest shares of elderly people in Europe and this proportion is expected to grow further.^25^ As most of 15 000–70 000 annual influenza-related deaths in Europe are registered in older adults,^11^ promoting SIV in this target population must be seen as a part of healthy ageing. We then demonstrated that the frequency of MOSIV differed by target vaccine, ranging from 5.0% to 23.3% in pneumococcal and COVID-19 cohorts, respectively, and some socio-structural factors, namely sex, age and nationality, were generally associated with MOSIV. Finally, we found that the co-administration of SIV with other recommended vaccines was relatively low, and this probably contributed to the observed magnitude of MOSIV.

To our knowledge, this is the first study to explore MOSIV in the context of visits for other recommended vaccines. Despite differences in methodological approaches, our results are in line with those of previous studies^9^^,^^26^ in showing that MOSIV occurs frequently. A recent survey^27^ of 29 482 US adults (21.1% of whom were aged ≥65 years) reported that 4.5%, 19.7% and 67.6% of older adults had received SIV only, COVID-19 vaccination only and both vaccines, respectively, while the remaining 8.2% had not received any vaccine. The proportion of MOSIV in the US survey^27^ resembles our estimate, despite the difference in healthcare systems.

We observed that SIV receipt was generally lower among younger and foreign-born older adults. Indeed, older age is probably the best-known positive predictor of SIV receipt.^28^^,^^29^ Analogously, many immigrant populations lack access to preventive care services.^30^ In this regard, Fabiani et al.^31^ reported that immigrants had a significantly lower likelihood (aOR 0.78; 95% CI: 0.68–0.90) of receiving SIV than Italians. Conversely, as emerged from a systematic review by Kini et al.,^32^ the association between sex and SIV uptake is controversial, while men are more likely than women to accept SIV, the actual SIV receipt may be higher in women than in men. This discrepancy might be explained by women’s greater use of preventive healthcare services and more frequent physician visits.^32^ In our study, the effect of sex on SIV receipt was significant only in the COVID-19 cohort, and the crude association would suggest a slightly higher (77.6% vs. 75.9%) vaccine receipt in male adults. However, the adjusted estimate showed a switch in direction, with men having 62% lower odds of receiving SIV, indicating major confounding by age and nationality. Furthermore, SIV receipt among both men and women depended on both age and place of birth, with younger and foreign-born men showing a lower SIV receipt. A similar finding was reported in a Spanish study,^33^ which revealed no between-sex difference in the 65–69-year age group and a significantly higher SIV uptake in males aged ≥70 years. However, other unmeasured factors, such as the presence of underlying health conditions or multimorbidity, may have contributed to the observed differences. Indeed, the presence of chronic conditions is a well-known predictor of SIV.^29^ In turn, the prevalence of co-morbidities in the Italian older adults may differ by age, sex and immigrant background.^34^ Future predictive models should also adjust for multimorbidity and/or other indicators of health status.

We found that one of the primary drivers of MOSIV was a suboptimal rate of vaccine co-administration, especially with regard to COVID-19 mRNA vaccines. Vaccine co-administration is probably the most efficient way to promote vaccination uptake, reduce the number of consultations and their associated costs, foster compliance with official recommendations, ensure the timely administration of vaccines according to the recommended schedules and adopt and administer newly licensed vaccines.^35^ One of the reasons for the suboptimal rate of concomitant SIV and COVID-19 vaccinations could be the newness of the latter. Evidence of this is the fact that the rate of co-administration of SIV and pneumococcal vaccines, which have been available for decades, was relatively high. Although several Italian and international Public Health Authorities have explicitly asserted the feasibility of COVID-19 vaccine co-administration,^10^ concomitant vaccination is only slowly becoming a common practice.^35^ It seems that the potential risk of increased reactogenicity is the main barrier to increasing co-administration rates.^10^^,^^35^ Experimental evidence, however, suggests^36^^,^^37^ that the available mRNA COVID-19 vaccines can be safely co-administered with SIV. It should also be borne in mind that, if vaccines were administered separately, transient adverse reactions would be reported at each visit, and the total cumulative number of transient adverse events would probably be greater than after the co-administration of two vaccines. Patients and healthcare providers should always consider vaccine co-administration, unless it is specifically contraindicated in the summary of product characteristics or discouraged by scientific evidence.^35^^,^^38^ Indeed, among all available adult vaccines, adjuvanted HZ vaccine should be co-administered only with non-adjuvanted SIVs.^39^ We believe that the most compelling future research should focus on the reasons for not co-administering SIV with other recommended vaccines from the perspectives of both the healthcare provider and the patient.

This study displays some important limitations, which arise from the structure and content of the vaccination registry used. First, as the vaccination registry contains a limited amount of information, some potential predictors of MOSIV (e.g. income, education, birth country of foreign-born individuals, presence of co-morbidities) were unavailable. For the same reason, the multivariable models may have suffered from the omitted-variable bias. Second, like all registry-based studies, the present one relies on the quality of data entry. Although GPs are incentivized to register each SIV administered for reimbursement purposes, we cannot exclude the possibility that some vaccinations were not registered. Third, the missed SIV administrations described represent only a part of MOSIV, since any visit (i.e. not only vaccination-related) to a healthcare provider by an eligible individual may constitute a MOSIV.^40^ In this study, by contrast, the denominator was the entirety of subjects who received other recommended vaccines. We believe that this share of MOSIV is the easiest to address, since it is unlikely to include subjects who are totally opposed to vaccinations. Fourth, the reasons for MOSIV could not be investigated. It is likely that most MOSIV were ascribable either to vaccine hesitancy^10^^,^^21^ or to the unavailability of SIV doses (e.g. at the very beginning/end of the SIV campaign, which is why the models were adjusted for the months of target vaccine administration). Finally, the study covers a local dataset and therefore our results may be not fully transferrable to other realities.

In conclusion, this study showed that a sizeable proportion of Italian older adults who received pneumococcal, HZ and, especially, COVID-19 vaccines during last winter did not receive the 2022/23 SIV, which is free of charge and recommended for all older adults. These unvaccinated individuals represent that part of MOSIV which is probably the easiest to address. The observed proportion of MOSIV was subject to some social inequalities, which may be addressed through tailored health promotion interventions. Recipients of COVID-19 vaccines, younger and foreign-born individuals, especially males, were generally at greater risk of MOSIV. Relatively low rates of co-administration of SIV with other recommended vaccines contribute to MOSIV. From a practical standpoint, we suggest that (i) local authorities should ensure an uninterrupted supply of SIV, implement active personalized SIV reminders and enhance alternative vaccination settings (e.g. pharmacy); (ii) vaccinating physicians should actively offer SIV at every outpatient or inpatient visit and during the entire influenza season; (iii) all physicians should have access to electronic vaccination registries; (iv) vaccinating physicians should make an extra effort to promote and actively offer SIV to younger and immigrant men at each visit; and (v) continuous medical education programmes should cover the topic of concomitant vaccine administration, which should be always considered.

## Data Availability

The data underlying this article may be requested from the corresponding author upon reasonable request and formal approval by the data holder (Local Health Unit 3, Genoa, Italy). Key points Missed opportunities for seasonal influenza vaccination (MOSIV) contribute to low vaccine uptake. MOSIV among adults who receive other recommended vaccines are the easiest to address. 20% of older adults who received other target vaccines missed their influenza vaccine. MOSIV were largely driven by suboptimal vaccine co-administration rates.
